# A Study of Surgical Cases During Earthquake Disaster in A Medical College

**DOI:** 10.31729/jnma.4063

**Published:** 2019-02-28

**Authors:** K.C Kanchan, Raj Kumar Thapa, Sanubhai Khadka, Damodar Paudel

**Affiliations:** 1 Department of General Practice and Emergency Medicine, Kathmandu Medical College Teaching Hospital, Sinamangal, Kathmandu, Nepal; 2 Department of Medicine, Shree Birendra Hospital, Chhauni, Kathmandu, Nepal; 3 Department of Medicine, Nepal Police Hospital, Kathmandu, Nepal

**Keywords:** *disaster*, *earthquake*, *Nepal*, *surgery*

## Abstract

**Introduction:**

An earthquake is an intense shaking of earth's surface which is caused by movements in earth's outermost layer. The earthquake of 25^th^ April 2015, with a magnitude of 7.8 richter scale with its major aftershock on 12^th^ May 2015 of 7.3 richter scale claimed around 8,962 lives across several districts of Nepal with 22,302 injuries. In this study we tried to figure out various surgical cases and the surgical procedures performed in a tertiary care hospital during an earthquake disaster.

**Methods:**

This study was a descriptive cross-sectional study of hospital data on all admitted surgical cases during an earthquake disaster. A total of 238 earthquake victims brought to emergency department of Kathmandu Medical College Teaching Hospital, a tertiary care center, from 26th April 2015 to 7^th^ Jun 2015, for the period of 42 days were included. Those brought dead and discharged after primary treatments were excluded. Data obtained were entered and analysed in Microsoft Excel 2010.

**Results:**

Among 238 patients enrolled, 122 (51%) were male and 116 (49%) female with male to female ratio of 1.05:1. Age group (31-60 years) with an average age of 45 years were encountered most frequently 110 (46%) with the maximum number of patient burden from Sindhupalchowk district 80 (33.6%). Orthopedic surgery 185 (76%) appeared to be the most frequent followed by neurosurgery, plastic surgery, general surgery and dental surgery.

**Conclusions:**

In natural disaster like earthquakes, traumatic injuries are very common and thereby various surgical procedures especially ortho-plastic are the domain of treatment modalities. Disaster preparedness and combined surgical team effort needs to be focused to reduce both mortality and morbidity.

## INTRODUCTION

Nepal is the 11^th^ most earthquake prone country in the world in terms of seismic vulnerability. In the history of Nepal, the earthquake of 25^th^ April 2015, with a magnitude of 7.8 richter scale and its major aftershock on 12^th^ May 2015, with 7.3 richter scale are the most devastating. It claimed 8,962 lives across several districts with 22,302 injuries.^1–4^

Reports from 2006 to 2016 that described 10 earthquakes worldwide found that between 600 and 220,000 people were killed per event.^5^ The impact of earthquakes has been reported to the highest in Asia, with China and Pakistan accounting for 40% of all earthquake related mortality.^6^ Trauma is the most common cause of mortality and morbidity through earthquake.^7^ Major surgery defined as an operation involving a considerable hazard or risking of life whereas minor surgery is a simple operation not considered to involve a risk to life.^8^

The aim of this study is to estimate various surgical cases and the surgical procedures performed during an earthquake disaster.

## METHODS

A descriptive cross-sectional study was done utilizing hospital data on all admitted surgical cases during an earthquake disaster. Earthquake victims of all age groups, a total of 238 patients brought to emergency of Kathmandu Medical College Teaching Hospital (KMCTH), a tertiary care hospital, from 26^th^ April 2015 to 7^th^ Jun 2015, for a period of 42 days were enrolled. Those patients who underwent various surgical procedures after admission in respective surgical departments were included and those brought dead and discharged after primary treatments were excluded.

Data from register book were retrieved after ethical approval from Institutional Review Committee of KMCTH. Standardized questionnaire developed by the researcher was used to obtain clinical and socio-demographic characteristics of the patients after getting informed consent. Depending upon the surgical procedure performed, cases were categorized into either major or minor surgery. Data obtained were entered and analysed in Microsoft Excel 2010. The descriptive statistical analysis was done.

## RESULTS

Among 238 patients enrolled, 122 (51%) were male and 116 (49%) female with male to female ratio of 1.05:1. Age group (31-60 years) with an average age of 45 years were encountered most frequently 110 (46%) with the maximum number of patient burden from Sindhupalchowk 80 (33.6%) and Kathmandu 40 (16.8%) districts (Table 1).

**Table 1.** Distribution of patients in districts.

Districts	n (%)
Kathmandu	40 (16.81)
Lalitpur	8 (3.36)
Bhaktapur	32 (13.45)
Sindhupalchowk	80 (33.61)
Rasuwa	10 (4.20)
Gorkha	11 (4.62)
Dhading	9 (3.78)
Nuwakot	8 (3.36)
Kavrepalanchowk	16 (6.72)
Ramechhap	10 (4.20)
Dolakha	8 (3.36)
Lamjung	1 (0.42)
Solukhumbu	1 (0.42)
Sankhuwasabha	1 (0.42)
Bardiya	1 (0.42)
Saptari	1 (0.42)
Sarlahi	1 (0.42)
Total	238 (100)

**Figure 1.**
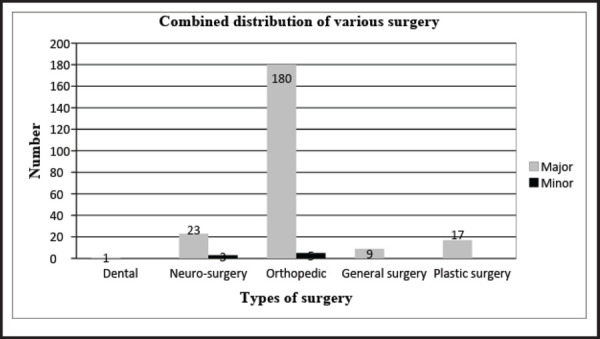
Combined distribution of various surgery.

In overall surgical procedures, orthopedic surgery appeared to be the most frequent followed by neurosurgery, plastic surgery, general surgery and dental surgery respectively (Figure 1).

Out of various orthopedic surgery performed, open reduction and internal fixation (ORIF) was the most frequent major surgery 111 (61%) with the total burden of 185 (76%) (Table 2).

**Table 2.** Distribution of major orthopedic surgery.

Major operation	n (%)
Amputation	3 (1.7)
External fixation	10 (5.6)
Internal fixation	111 (61.7)
LCDCP^*^	10 (5.6)
DHS^†^	2 (1.1)
Arthrotomy	2 (1.1)
Traction	2 (1.1)
Debridement	25 (13.88)
CR^‡^	3 (1.6)
K-wire fixation	3 (1.6)
TENS^§^	2 (1.1)
Secondary closure	2 (1.1)
Spinal decompression	1 (0.55)
TBW^||^	2 (1.1)
Relocation	2 (1.1)
Total	180 (100)

* Limited contact dynamic compression plate.

† Dynamic hip screw.

‡ Closed reduction.

§ Transcutaneous electrical nerve stimulation.

|| Tension band wiring.

**Figure 2.**
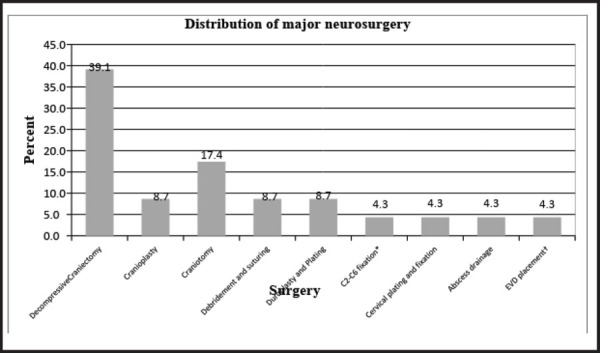
Distribution of major neurosurgery.

^*^Cervical vertebra 2^nd^ to 6^th^ fixation.

^†^External ventricular drain placement.

**Figure 3.**
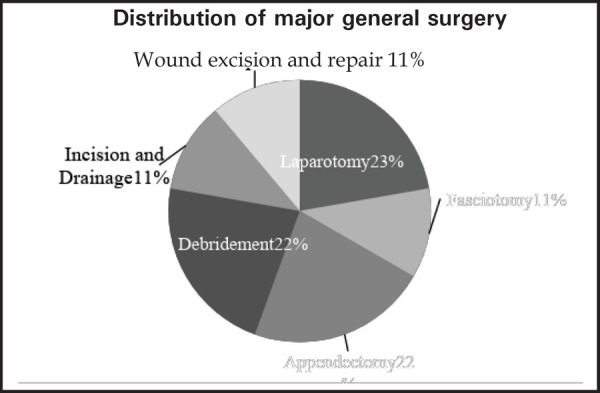
Distribution of major general surgery.

Likewise, decompressive craniectomy was the most frequent major neurosurgical procedure 9 (39%) (Figure 2) and debridement with split skin graft (SSG) 13 (76%) in plastic surgery. In general surgery, laparotomy and debridement accounted for 2 (22%) each (Figure 3).

## DISCUSSION

In our study population, almost equal number of male 122 (51%) and female 116 (49%) were encountered with the most frequent age group of 31-60 years. In a similar study by Thapa SS et al,^9^ majority of population (61%) were in the age group 16-60 years. Likewise, in the prospective hospital based study by Giri S et al,^10^ around 74% population were of the age group 15-65 years and in the earthquake disaster relief mission in Nepal by Singapore Armed Forces,^11^ there were 77.9% adults.

Out of 35 seriously earthquake stricken districts maximum number of patient burden in our hospital were from Sindhupalchowk district 80 (33.6%) followed by Kathmandu 40 (16.8%) and then Bhaktapur 32 (13.4%). In the similar Dhulikhel hospital based study by Giri S et al,^10^ bulk of the patients were also from Sindhupalchowk district 55%, possibly because of nearby location of the hospital.

In epidemiology of traumatic injuries from earthquake by Ramirez M et al,^12^ it is stated that according to human/individual factors, built environment and the seismic/geologic factors the number and type of injuries caused by earthquake vary. However, most of the time earthquake related injuries are orthopedic in nature, commonly fractures that of the long bones.^13^ In our study also, out of various surgical cases, majority were related to orthopedics 77.7% and remaining 22.3% were non-orthopedics in nature (neurosurgery 10.9%, plastic surgery 7.1%, general surgery 3.7% and dental surgery 0.04%.

Similar results were also seen in the study by Thapa SS et al,^9^ where 81% were orthopedics and 19% non-orthopedics, commonly involving lower limb fractures, around 77%. Likewise, in a review earthquake related article by MacKenzie JS et al,^13^ suggested 87% cases that of orthopedics, nearly 65% of them were fractures commonly involving the lower limbs. It was also supported by related article by Shrestha JM et al,^14^ on role of plastic reconstructive surgery during Nepal earthquake 2015.

In orthopedic surgery, open reduction and internal fixation (ORIF) 111 (61.7%) was the commonest surgery performed followed by debridement 25 (13.9%). In contrary, external fixation and debridement constituted the bulk of the cases in a similar study by Thapa SS et al,^9^ possibly because of the type of fracture cases received in their center. In earthquake related disaster situations, external fixation is vital for proper management of fractures and soft tissue stabilization and the ratio of external fixation to ORIF mainly rely on the time of arrival of disaster response team at the earthquake site, supported by McIntyre T et al,^15^ on Haiti earthquake and Rajpura A et al,^16^ on Pakistan earthquake. However, in a review article by MacKenzie et al,^13^ on average 12% of fractures were stabilized by external fixation and the use of external fixation vary from less than 2% to more than 30%.

In Neurosurgery, decompressive craniectomy 9 (39.1%) was the commonest surgery performed in our study. Here it appears to be the second most common surgery after orthopedics in post earthquake scenario. In a similar study of 1999 Taiwan earthquake,^17^ around 30% of people died from head injuries and after lower limb injuries head injuries were the second most frequent in 2008 Sichuan earthquake.^18^

Among all plastic surgical cases in our study, debridement with split skin grafting (SSG) accounted 13 (76.5%). Similarly in a study by Shrestha JM et al,^14^ 22% of the total operations were for soft tissue injury and the most common surgery was SSG 39 (34%). Skin grafting after soft tissue injury was a common procedure ranging from 22% to 43%, Rajpura A et al,^16^ and Clover AJ et al.^19^ Similar findings in studies by Zhang J et al,^20^ and Wolf Y et al.^21^

For non-salvageable limbs, amputation rate in our study was 3 (1.7%). It was supported by various studies where amputation rates ranges between 0.4 to 11%, Phalkey R et al,^22^ and Yang C et al.^23^

In General surgery, laparotomy and debridement accounted for 2 (22.2%) each in our study. In one series from Haiti earthquake, gastrointestinal cases were only 2% of the total operative volume and bulk occupied by plastic surgery and orthopedics.^24^ In contrast, in the study of patterns of abdominal injury in thousands of earthquake victims of Wenchuan earthquake, abdominal injury was often accompanied with multiple injuries and around 32% had earthquake-related abdominal injury where spleen was the most commonly injured abdominal organ (18%), showing various factors define injury patterns.^25^

In our study only one documented mortality 1 (0.4%) in total admitted surgical cases. In an earthquake, the number of injured far exceeds the death toll and on average injury to mortality ratio stands at around 3:1.^26,27^ In the study done by Thapa SS et al ratio was 2.5:1 and overall mortality of earthquake related injured hospitalized patients was 2.6%.^9^ In a similar study by Giri S et al the 90 days mortality was only 2%.^10^ However overall mortality among such can be as high as 8%.^28^ The limitations of our study being the data of a single referral center and no follow up visits study.

## CONCLUSIONS

In various earthquake related articles including ours, it is seen that ortho-plastic and allied surgical backup in hospitals should be focused to reduce both mortality and morbidity. Besides disaster preparedness, national policy, a systematic approach and a team effort is a must to be effective in such disastrous situations.
